# Percutaneous Disc Decompression with Nucleoplasty–Volumetry of the Nucleus Pulposus Using Ultrahigh-Field MRI

**DOI:** 10.1371/journal.pone.0041497

**Published:** 2012-07-25

**Authors:** Richard Kasch, Birger Mensel, Florian Schmidt, Wolf Drescher, Ralf Pfuhl, Sebastian Ruetten, Harry R. Merk, Ralph Kayser

**Affiliations:** 1 Clinic and Outpatient Clinic for Orthopedics and Orthopedic Surgery, University Medicine Greifswald, Greifswald, Germany; 2 Department of Diagnostic Radiology and Neuroradiology, University Medicine Greifswald, Greifswald, Germany; 3 Department of Orthopedic and Trauma Surgery, RWTH Aachen University, Aachen, Germany; 4 Leibnitz Institute for Farm Animal Biology, Dummerstorf, Germany; 5 Department of Spine Surgery and Pain Therapy, Center for Orthopedics and Traumatology, St. Anna-Hospital Herne, Herne, Germany; University of Louisville, United States of America

## Abstract

**Purpose:**

To evaluate changes in nucleus pulposus volume as a potential parameter for the effects of disc decompression.

**Methods:**

Fifty-two discs (T8 to L1) were extracted from 26 pigs and separated into thoracic (T8 to T11) and thoracolumbar discs (T12 to L1). The discs were imaged using 7.1 Tesla ultrahigh-field magnetic resonance imaging (MRI) with acquisition of axial T2-weighted turbo spin-echo sequences for determination of baseline and postinterventional nucleus pulposus volumes. Volumes were calculated using OsiriX® (http://www.osirix-viewer.com). After randomization, one group was treated with nucleoplasty, while the placebo group was treated with an identical procedure but without coblation current. The readers analyzing the MR images were blinded to the kind of procedure performed. Baseline and postinterventional volumes were compared between the nucleoplasty and placebo group.

**Results:**

Average preinterventional nucleus volume was 0.799 (SD: 0.212) ml. Postinterventional volume reduction in the nucleoplasty group was significant at 0.052 (SD: 0.035) ml or 6.30% (p<0.0001) (thoracic discs) and 0.082 (SD: 0.042) ml or 7.25% (p = 0.0078) (thoracolumbar discs). Nucleoplasty achieved volume reductions of 0.114 (SD: 0.054) ml or 14.72% (thoracic) and 0.093 (SD: 0.081) ml or 11.61% (thoracolumbar) compared with the placebo group.

**Conclusions:**

Nucleoplasty significantly reduces thoracic and thoracolumbar nucleus pulposus volumes in porcine discs.

## Introduction

Magnetic resonance imaging (MRI) has gained an important role as a noninvasive tool in biomedical research. No harmful side effects are known, and its excellent soft tissue contrast makes it the imaging procedure of choice for examinations of the spine [1], [2]. While 1.5 and 3 Tesla MRI have become established in clinical routine [3], high-resolution 7 Tesla ultrahigh-field MRI is becoming increasingly available for answering more specialized questions [4]. Data on disc morphology and the effects of intradiscal therapy are still limited. MRI is well suited for providing such data, allowing measurement of intradiscal volume [5]–[6].

In the past a variety of different intradiscal procedures were used for treating symptomatic disc prolapse [7]–[10], many of which have now been abandoned [11]. Various studies have reported the clinical results of these treatments [8] [10] [12]–[14], and the effectiveness of some procedures has been demonstrated in high-quality studies [7] [10] [9] [15]. For many procedures, however, the mechanism of action remains to be demonstrated in an experimental setting.

More than 10 years ago, in July 2000, nucleoplasty was approved in the USA by the Food and Drug Administration (FDA) as a treatment for symptomatic disc prolapse [16]. Since then several valid studies have demonstrated its clinical effectiveness in treating the lumbar spine [16], and it is now considered safe and reliable [17]. Chen et al. experimentally demonstrated that nucleoplasty works by reducing pressure [18], and its histological effect on disc tissue has been characterized as well [19]–[20]. Experience with other intradiscal procedures suggests that the clinical effectiveness of nucleoplasty is due to its volume-reducing effects [21]. To the best of our knowledge, a study experimentally investigating volume reduction after nucleoplasty has not been published in the English literature.

This study investigated the question of whether nucleoplasty has volume-reducing effects on biomechanically different spinal segments in pigs - the thoracic and thoracolumbar spine [22]–[23] - and whether these effects can be demonstrated by volumetry of the nucleus pulposus using in vitro data sets acquired by 7 Tesla MRI.

## Materials and Methods

### Specimens

Our study included 53 ex vivo discs (T8- L1) from 26 freshly slaughtered “German native breed” pigs (mean age, 12.7 months; range, 4–54 months). We differentiated between thoracic (T8 to T11) and thoracolumbar junction discs (T12 to L1).

Discs were assigned randomly to either the nucleoplasty group or the placebo group, and the experiment was performed within 24 hours of slaughter. Discs showing damage (from slaughter, transport, etc.) on gross inspection or MRI were excluded from analysis (n = 1).

### Imaging

**Figure 1:**
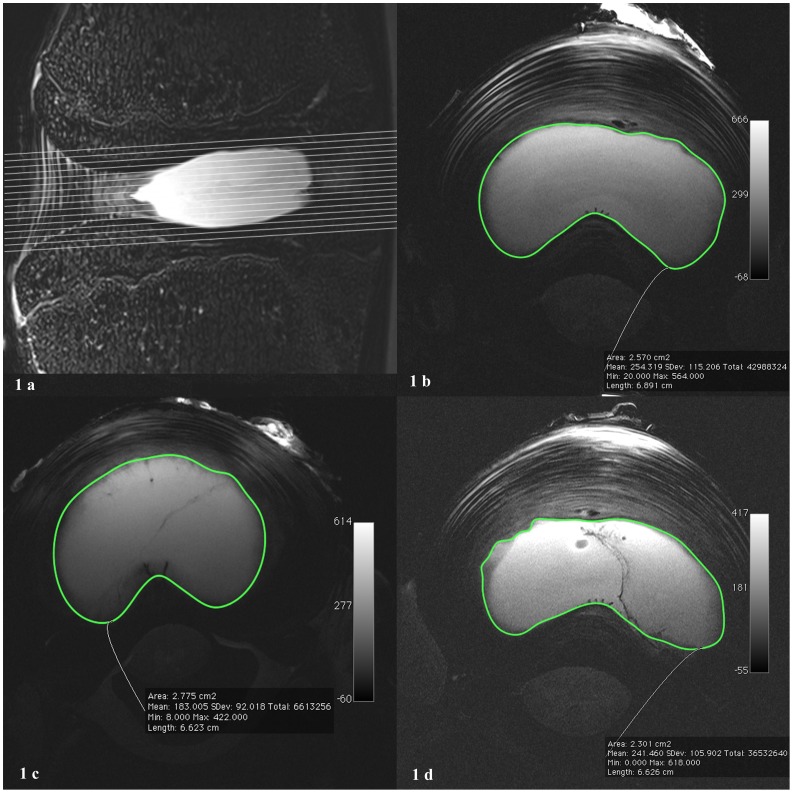
T2-weighted sagittal and axial images of ex vivo discs of pigs, depicting the hyperintense nucleus pulposus and the surrounding inhomogeneous hypointense anulus fibrosus. a: Sagittal image of lumbar motion segments showing axial stack. The axial images were acquired without gaps, parallel to the disc. b: Preoperative image. The green line outlines the nucleus pulposus area for volume calculation. c: Postinterventional image of the placebo group. Visible coblation channel at the 7–8 o’clock position on the left. d: Postinterventional image of nucleoplasty group. Visible coblation channel at the 4–5 o’clock position on the right; the channel is hypointense in the nucleus pulposus and hyperintense in the anulus fibrosus.

Imaging was performed on a 7.1 Tesla MR imager (ClinScan, Bruker Bioscan GmbH, Ettlingen, Germany). Discs were placed on 1-channel surface coils with the ventral side down and examined. Before treatment, a mark was placed on each disc to match preoperative and postoperative disc positions relative to the coil. The entire nucleus pulposus was imaged (Fig. 1A) using axial, gapless, T2-weighted turbo spin-echo sequences (Fig. 1B-D) (repetition time (TR), 2000 ms; echo time (TE), 42 ms; slice thickness, 0.7 mm; field of view (FOV), 45×45 mm; 512×512 pixels). The acquisition time was 7∶20 min.

### Volumetry

MR images were viewed and processed using the OsiriX® software (version 3.6.1, 32-bit, http://www.osirix-viewer.com) [24]. Volumetry of the nucleus pulposus was performed semiautomatically. A radiologist manually outlined the junction of the nucleus pulposus and the anulus fibrosus, first cranially and then caudally, in the axial image stack (Fig. 1B-D). The software then generated the missing outlines between the cranial and the caudal slices, and each slice was afterwards corrected manually. OsiriX® was used to calculate the nucleus pulposus volume by multiplying all outlined areas of the slices with the slice thickness. Volumes were measured and calculated in the same way before and after nucleoplasty or placebo treatment (Fig. 1A-D).

### Nucleoplasty

We used the ArthroCare System 2000 (Arthrocare Deutschland, Remscheid, Germany) with control unit, foot switch, and Convenience Pack (DLR SpineWand and sterile 17-gauge Crawford needle (6“) with mandrin). Appropriate to the manufacturer’s instructions, the ex vivo coblation current, in the nucleoplasty group (Fig. 1D), was applied in 6 positions for 10 sec each to create 6 channels with an application field of 360°. In the placebo group (Fig. 1C) the identical procedure was performed but without application of current. Discs were randomized to nucleoplasty or placebo treatment. The volumes of the discs treated with and without application of coblation current were then compared to determine the effectiveness of nucleoplasty.

### Data Selection

A total of 52 discs were included. Unpaired samples – randomly selected, independent value pairs – were assigned to one of two groups: thoracic discs and thoracolumbar junction discs, which have different biomechanical characteristics. Independent value pairs were selected randomly to minimize the potential for systematic bias (global disc disease in an individual pig, for example). Thus, only one functional lumbar spinal unit (vertebra-disc-vertebra) (T8/T9 and T9/T10) was considered per spine. The two groups (therapy versus placebo group) were formed using the SAS randomization program. For the thoracic spine, 18 discs were assigned to the therapy group and 18 to the placebo group. For the thoracolumbar spine, each group was assigned 8 discs.

### Statistics

The data were assessed with the Wilcoxon test for independent (nonparametric) samples using SAS 9.1 TS (XP_PRO Windows NT Server, Cary, North Carolina, USA). Data are given as absolute values and standard deviations. Calculated differences were considered statistically significant at p<0.05.

## Results

There were no complications during the interventions. All porcine discs were found to be normal, showing no abnormalities or degenerative changes. In both postoperative groups we were able to track the placebo/nucleoplasty channel created in the center of the disc (Fig. 1C-D). The average nucleus volume for all 52 examined discs was 0.799 (SD: 0.212) ml.

**Figure 2:**
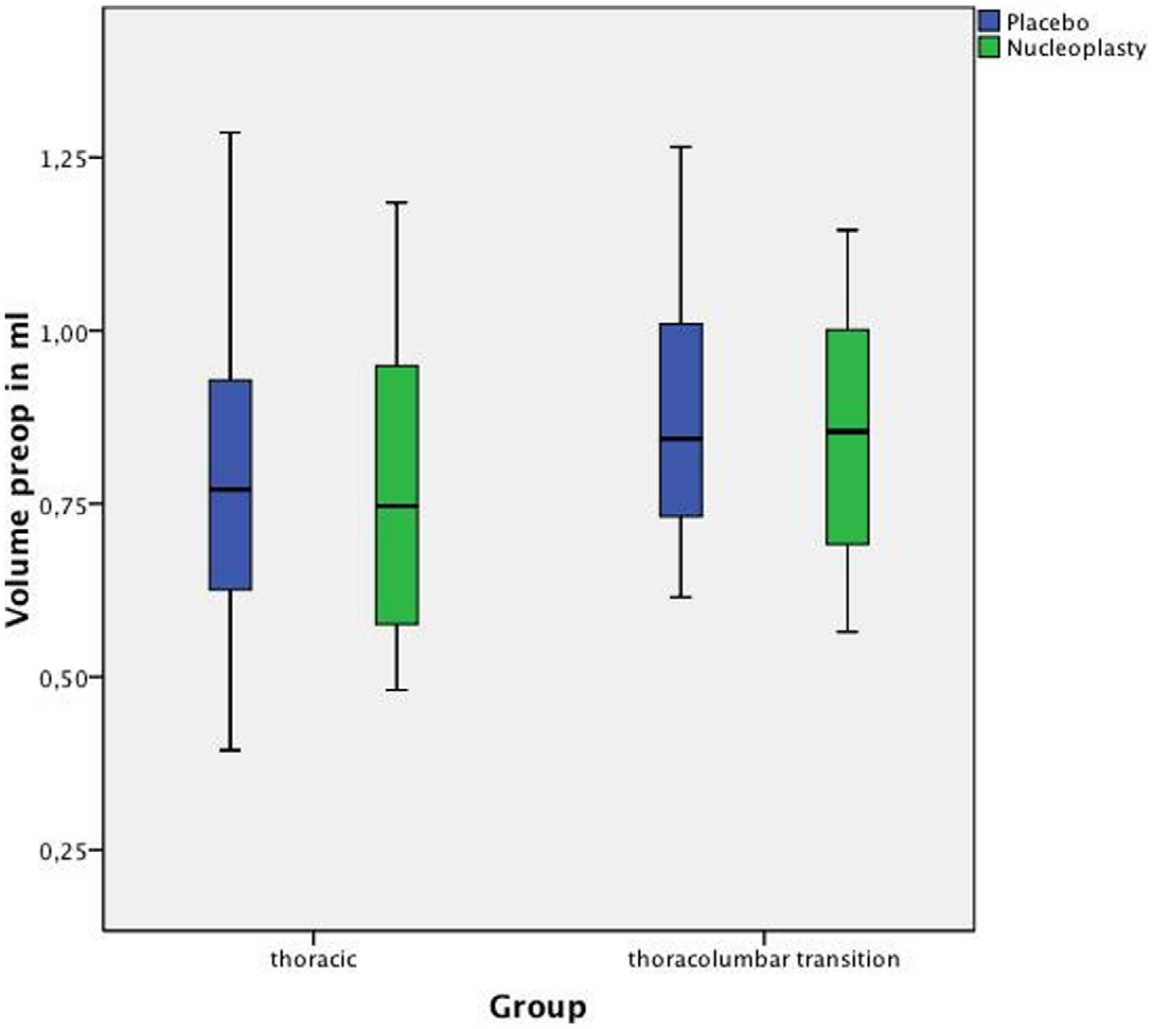
Initial nucleus pulposus volumes in the independent thoracic (placebo group n = 18, nucleoplasty group n = 18) and thoracolumbar (placebo group n = 8, nucleoplasty group n = 8) groups in a box-whisker plot, presenting the 25% (lower box end), 50% (marking in the box) and 75% quartiles (lower and upper box end). The whiskers represent the smallest and largest values in the 1.5× interquartile range. The group differences were not significant - p = 0.130 and p = 0.461.

**Figure 3:**
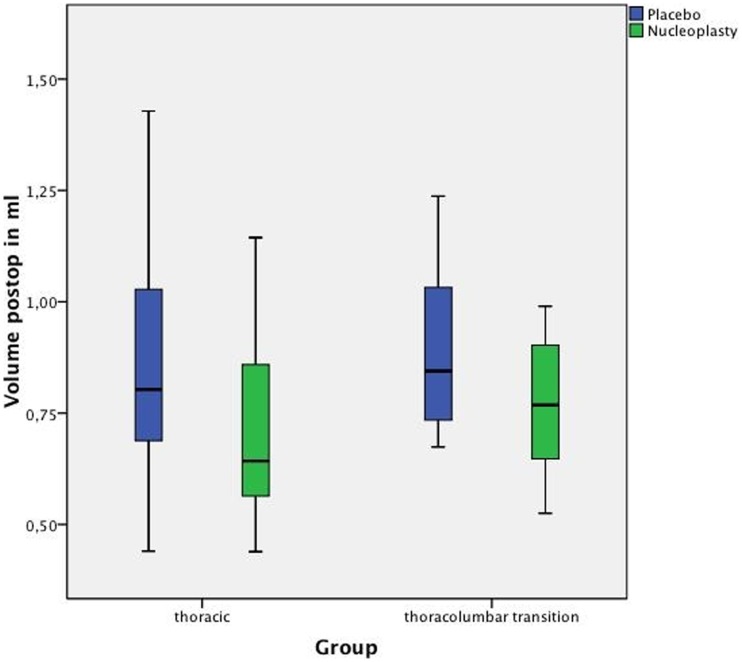
Postoperative volumes in the independent placebo and nucleoplasty groups for the two spinal segments investigated.

The results for thoracic discs were as follows. The average baseline volume was 0.754 (SD: 0.203) ml in the nucleoplasty group (n = 18) and 0.786 (SD: 0.219) ml in the placebo group (n = 18), showing no statistically significant difference (p = 0.130) (Fig. 2). Thoracic discs treated with nucleoplasty showed a significant nucleus pulposus volume reduction (pre- versus postprocedure) of 0.052 (SD: 0.035) ml or 6.30% (p<0.0001). Placebo-treated thoracic discs showed a significant volume increase (due to instrument manipulation) of 0.062 (SD: 0.053) ml or 8.42% (p = 0.0002) (Table 1, Fig. 3).

**Table 1:** Summary of intradiscal volume changes in the thoracic (n = 36) and thoracolumbar (n = 16) spine: nucleoplasty versus placebo.

Cohort	n	Absolute volume change				Relative volume change	
Thoracic discs		Nucleoplastygroup (n = 18)	P	Placebo group(n = 18)	p	Nucleoplastygroup (n = 18)	Placebo group(n = 18)
Total	36	−0.052 ml(SD: 0.035)	<0.0001	0.062 ml(SD: 0.053)	0.0002	−6.30%	8.42%
Thoraco-lumbar discs		Nucleoplasty group(n = 8)	P	Placebo group(n = 8)	p	Nucleoplasty group(n = 8)	Placebo group(n = 8)
Total	16	−0.082 ml(SD:0.042)	0.0078	0.011 ml(SD:0.082)	0,547	−7.25%	4.36%

**Figure 4:**
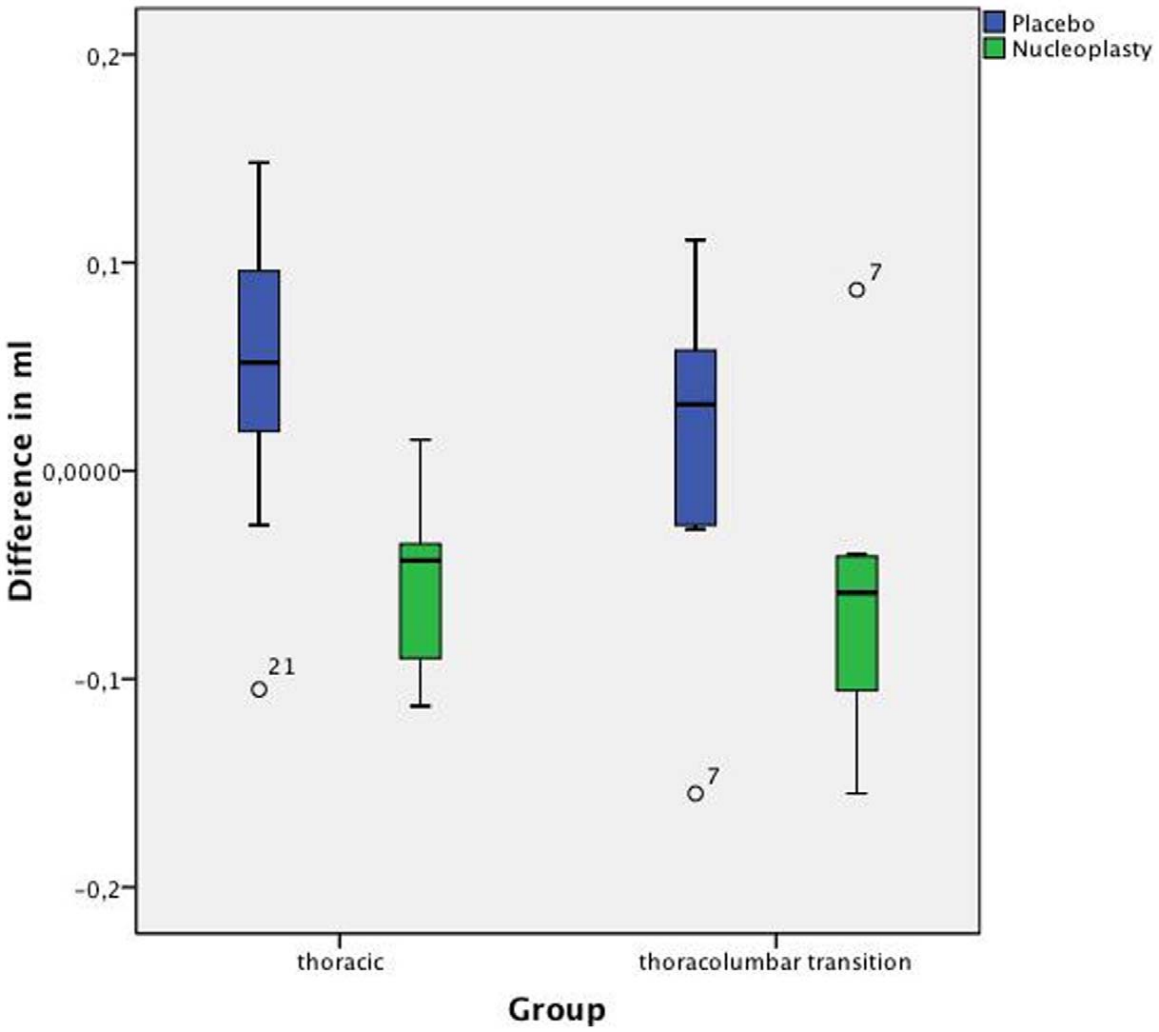
Differences between initial and postoperative volumes for placebo and nucleoplasty groups. Comparison of the values of the thoracic group and thoracolumbar group shows a volume increase in the placebo group and a volume reduction in the nucleoplasty group (_°_- markers denote “mild” outliers within the 1.5× and 3× interquartile ranges).

In the thoracic group, nucleoplasty decreased nucleus volume by 0.114 (SD: 0.054) ml or 14.72% compared with the placebo group (Table 2, Fig. 4).

**Table 2:** Analysis of intradiscal volume changes in the thoracic spine for independent pairs (n = 18).

Thoracic discs	Average (mean)	Median	SD	Range	Interquartile range	p
Δ V absolute in ml	0.114	0.093	0.054	0.207	0.076	<0.0001
Δ relative in %	−14.725	−15.090	6.747	25.980	6.670	<0.0001

The average baseline volume of the larger thoracolumbar junction nuclei was 0.850 (SD: 0.200) ml for the nucleoplasty group (n = 8) and 0.881 (SD: 0.214) ml for the placebo group (n = 8), showing no significant difference (p = 0.461) (Fig. 2). For the thoracolumbar junction group as well, a significant postnucleoplasty volume reduction (pre- versus postprocedure) of 0.082 (SD: 0.042) ml or 7.25% (p = 0.0078) was demonstrated. In the placebo group, a nonsignificant volume increase (due to instrument manipulation) of 0.011 (SD: 0.082) ml or 4.36% (p = 0.547) was measured (Table 1, Fig. 3).

In the thoracolumbar junction group, nucleoplasty decreased nucleus volume by 0.093 (SD: 0.081) ml or 11.61% compared with the placebo group (Table 3, Fig. 4).

**Table 3:** Analysis of intradiscal volume changes in the thoracolumbar spine for independent pairs (n = 8).

Thoraco-lumbar discs	Average (mean)	Median	SD	Range	Interquartile range	p
Δ V absolute in ml	0.093	0.115	0.081	0.264	0.073	<0.05
Δ relative in %	−11.610	−11.880	8.280	27.240	8.480	<0.05

## Discussion

Different studies have demonstrated the effectiveness of minimally invasive intradiscal procedures in treating symptomatic disc prolapse [7]–[8] [10] [12] [16], [25] [26]. In order to gain long-term acceptance, however, clinical treatment methods require more than simply evidence-based data evaluation. Their effectiveness must also be measurable in experimentally verifiable models [27]. For chemonucleolysis or percutaneous laser disc decompression (PLDD), with a level of evidence II-2 for short- and long-term pain relief, [8] the mechanism of action has now been clarified in an experimental setting [14] [28]–[31]. For nucleoplasty, however, before this study, this was not the case.

Coblation employs an electrolyte-rich medium to generate a plasma field of highly ionized particles with enough energy to break the molecular bonds in soft tissue, so that tissue is vaporized and escapes through the introducer needle [32]. It is a technology that has been successfully established in a number of therapeutic fields, not only those involving the musculoskeletal system [33]–[34].

The risk of indirect injury to nerve structures close to the discs has been discussed repeatedly. Temperatures of 60 to 65°C or even higher can be reached at a distance of 3–4 mm from the probe [35]. Considering that neurodegeneration begins at 45°C [36], this is not to be taken lightly. Several experiments, however, performed both in vivo and in vitro using discs from pigs and sheep, have demonstrated that any tissue damage produced by coblation will be confined to the plasma field surrounding the electrode [19]–[20] [36].

Some intradiscal procedures have proven to be more effective when they are restricted to discs that have not lost too much height or volume due to degeneration [37]. Intradiscal electrothermal therapy (IDET) offers, significant relief in one-half of chronic discogenic low back pain patients [10] and, has been demonstrated to be more effective than placebo only when height loss of the discs in the segment treated is less than 20% [38]. With height loss of 50% or more, no therapeutic effect can be demonstrated at all [14]. The efficacy of most intradiscal procedures is tied to a particular disc volume, limiting their usefulness in cases of height loss or disc degeneration. For this reason our study only included discs that were free of all signs of degenerative damage in the preinterventional MRI.

Chen et al. demonstrated experimentally that nucleoplasty involves a reduction in pressure within the disc [18]. Using three human cadavers they also showed that the intradiscal pressure reduction achieved by nucleoplasty on lower thoracic and lumbar discs was dependent on the degree of disc degeneration. Applying monopolar radiofrequency in an in vivo sheep model Podhaysky et al. also demonstrated an intradiscal pressure reduction that was still measurable 4 weeks later [37]. The demonstrated pressure effects are attributable to the reduction in nucleus pulposus volume we show in our study. To our knowledge, such quantitative volume effects have not been published before.

The mechanism of action of PLDD can be described as volume reduction – which also results in intradiscal pressure reduction [39]–[41]. Volumetry of organs and organ systems is widely used and well established in medical research and has found a prominent place in routine clinical examinations as well. MR data sets are used to outline the target volume manually, semiautomatically, or fully automatically. This, along with the remaining image parameters (slice thickness, gap), is used to calculate the volume. Volumetry is thus used in the diagnosis of neurodegenerative diseases, cardiac diseases, and stroke as well as in the planning and follow-up of operative and nonoperative cancer treatment [42]–[45].

Tissue vaporization with reduction of the nucleus pulposus volume appears to be the mechanism of action by which nucleoplasty reduces pressure in intact discs as used in our study. Chen et al. demonstrated this volume-reducing effect qualitatively by performing postoperative histological examinations [19] without providing any observations on the quantitative extent of ablation or its effects. Case et al. also showed a correlation between pressure and volume changes in the disc [21].

Our experimental data show that, in a placebo-controlled setting, nucleoplasty can be used to reduce the initial volume of nucleus pulposus by a statistically highly significant degree. We attribute the volume increase in the placebo group to the fact that the nucleus was pushed aside and compressed by insertion of the Spine Wand. Conversely, in the nucleoplasty group, the compressed volume was reduced with the coblation current.

Our study examined regions of the porcine spine that bear the closest anatomical resemblance to the human spine [46]–[48]. First we quantified the volume-reducing effects of nucleoplasty on the lower thoracic spine and on the thoracolumbar junction to the upper lumbar spine, corroborating this effect by comparison with the placebo group. We demonstrated that nucleoplasty reduced initial nucleus pulposus volumes and also that these volume reductions – (T8- T11 = 14.72%) and (T12 to L1 = 11.61%) – were significant compared to the placebo procedure. Although the porcine disc model we used is very similar to the human spine, our experimental results do not allow us to draw any conclusions regarding the volume reduction in humans. This is precluded because porcine and human discs are not fully identical and because we did not measure the amount of material removed.

While the thoracic discs of the thoracic spine were smaller than the discs of the thoracolumbar junction, the volume reduction achieved by nucleoplasty was similar in the two groups. It can be assumed that decompression involves the entire anulus fibrosus and not the herniated disc portion alone [28]. Intradiscal pressure decreases in proportion to volume reduction [21]. The proteins within the nucleus pulposus exert an osmotic force that produces a continual flow of water into the disc [30]. Nucleoplasty, like PLDD, denatures some of the proteins, thus reducing the reperfusion effect in a way that contributes to the long-term effectiveness of the procedure [19] [41].

### Conclusion

Our study demonstrates that nucleoplasty has a volume-reducing effect on the nucleus pulposus of the thoracic and thoracolumbar spine. While this effect was demonstrated in an experimental setting and remains to be verified in patients, our results suggest that the potential benefit likely to be achieved in the clinical setting is of interest and deserves to be pursued further.
